# Auditory conditioned stimulus presentation during NREM sleep impairs fear memory in mice

**DOI:** 10.1038/srep46247

**Published:** 2017-04-12

**Authors:** Ross J. Purple, Takeshi Sakurai, Masanori Sakaguchi

**Affiliations:** 1 Sleep and Circadian Neuroscience Institute, Nuffield Department of Clinical Neurosciences, University of Oxford, Sir William Dunn School of Pathology, Oxford, OX1 3RE, UK; 2 International Institute for Integrative Sleep Medicine, University of Tsukuba, 1-1-1, Tennodai, Tsukuba, Ibaraki, 305-8575, Japan

## Abstract

Externally manipulating memories by presenting conditioned stimuli (CS) during sleep is a new approach to investigating memory processing during sleep. However, whether presenting a CS during REM or NREM sleep enhances or extinguishes fear memory has not been clearly delineated. In this study, mice underwent trace fear conditioning consisting of an auditory CS paired with a foot shock, and the auditory CS was re-presented during subsequent REM or NREM sleep. Mice that received auditory cueing during NREM but not REM sleep showed impaired fear memory upon later presentation of the auditory CS. These findings have implications for the use of cueing during sleep and advance our understanding of the role of REM and NREM sleep in memory consolidation.

During memory consolidation, memories that are initially transient connections vulnerable to interference^1^ become reinforced and eventually transform into more stable, long-term networks. Sleep may play an active role in memory consolidation^2^ in humans^3^,^4^,^5^ and rodents^6^,^7^. Sleep can be broadly categorised as rapid eye movement (REM) sleep and non-REM (NREM) sleep, which are characterised by stark differences in global oscillatory network activity in the brain as seen by electroencephalography (EEG). Sleep has been postulated to enhance memory consolidation through these network oscillations, such as slow-wave activity (0.5–4 Hz) during NREM sleep^8^,^9^ and theta oscillations (4–7 Hz) during REM sleep^10^.

Recent literature reports that the consolidation of new memories can be manipulated during sleep. In a remarkable example of this phenomenon, Rasch *et al*.^11^ found that presenting humans with an olfactory cue during NREM sleep that was previously presented during the learning of an object location task enhanced memory consolidation. Subsequent studies report that memory consolidation is enhanced by presenting auditory stimuli during sleep^12^, with such cueing effective in both rats^13^,^14^,^15^ and mice^16^. In humans, this phenomenon appears to be specific to NREM sleep, with no effect of cueing typically observed during REM sleep^17^,^18^,^19^. To our knowledge, only one study has attempted cueing during REM sleep in rodents, which, contrary to previous findings, improved memory consolidation^15^. Also, inconsistencies in the effect of cueing during different sleep stages are seen in studies of emotionally salient memories, which report that cueing during NREM or REM sleep can enhance (NREM^13^,^16^,^20^; REM^15^), extinguish (NREM^21^,^22^), or have no effect (REM^18^,^19^) on memory. Therefore, how cueing affects emotional memories and the differential contributions of NREM and REM sleep to memory consolidation require further investigation.

Here, we examined how re-presenting an auditory conditioned stimulus (CS) during REM or NREM sleep affects the consolidation of a fear memory in mice. We believe this is the first study to directly compare the effect of cueing during NREM versus REM sleep in rodents. Based on previous findings from Barnes and Wilson^13^ and Rolls *et al*.^16^, who used a similar fear conditioning/NREM sleep intervention protocol, we hypothesised that presenting an auditory CS associated with a fearful stimulus during NREM sleep enhances consolidation of the fear memory.

## Results

**Figure 1:**
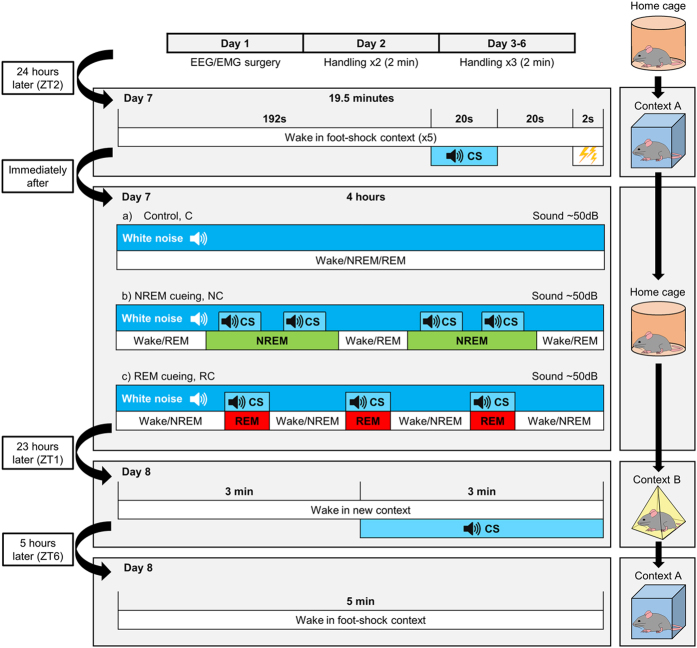
Experimental timeline. After implantation of EEG/electromyogram (EMG) electrodes, mice recovered for 6 days, during which they were habituated to gentle handling. On day 7, mice were transferred to a training context (context A) in which they underwent fear conditioning consisting of a 192-s acclimation, 20-s auditory CS, 20-s interval, and 2-s foot shock; this sequence was repeated five times. Mice were returned to their home cages and split into three groups for a 4-hour intervention involving continuous EEG/EMG recording. For control mice, white noise was played for the duration of the intervention. For NREM- and REM-cued mice, the auditory CS was presented for a total of 5 minutes, during either NREM or REM sleep, respectively. To account for differences in NREM and REM period lengths, CS presentation was allowed throughout REM periods in REM-cued mice but limited to a maximum of 30 seconds during NREM periods in NREM-cued mice. Background white noise was reduced during CS presentation so that for all three groups, the total sound pressure level was maintained at 50 dB throughout the intervention period. Twenty-three hours later, mice were placed into a novel context (context B) in which they underwent cued fear memory testing consisting of a 3-min acclimation and 3-min auditory CS. Mice were returned to their home cages. Five hours later, mice were placed in context A for 5 min for contextual fear memory testing. See methods for further details.

Mice were fear conditioned by presenting an auditory CS prior to delivery of a foot shock in a training context (context A; Fig. 1). The auditory CS was then re-presented over 4 hours of NREM sleep (NREM-cued group, NC; *n* = 10) or REM sleep (REM-cued group, RC; *n* = 10). Mice in the control group (C; *n* = 9) were played white noise during sleep. The next day, the auditory CS was presented to mice in a novel context (context B) to test for cued fear memory (i.e., association between the auditory CS and shock). Finally, the mice were returned to the training context (context A) to test for contextual fear memory (i.e., association between the context and shock; Fig. 1).

### Auditory cueing during sleep induced slight changes in sleep architecture and spectral power

**Figure 2:**
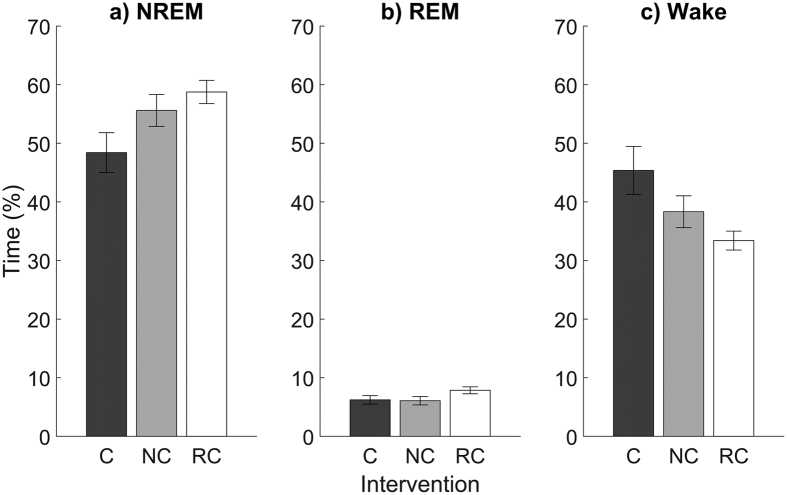
Time spent in (**a**) NREM sleep, (**b**) REM sleep, and (**c**) wake states during the 4-hour intervention for mice in the control (C), NREM-cued (NC), and REM-cued (RC) groups. Data are shown as mean ± SEM.

We first examined sleep physiology to determine the impact, if any, of presenting the auditory CS during sleep. NREM- and REM-cued mice showed a trend toward more NREM sleep and less total wake time, but these differences did not reach significance (Table 1, Fig. 2a–c). NREM-cued mice had a similar number of transitions between NREM, REM, and wake states during the 4-hour intervention compared with REM-cued and control mice, whereas REM-cued mice had significantly more REM-to-NREM transitions than control mice (Table 1). There were no other group differences in measures of sleep architecture.

**Figure 3:**
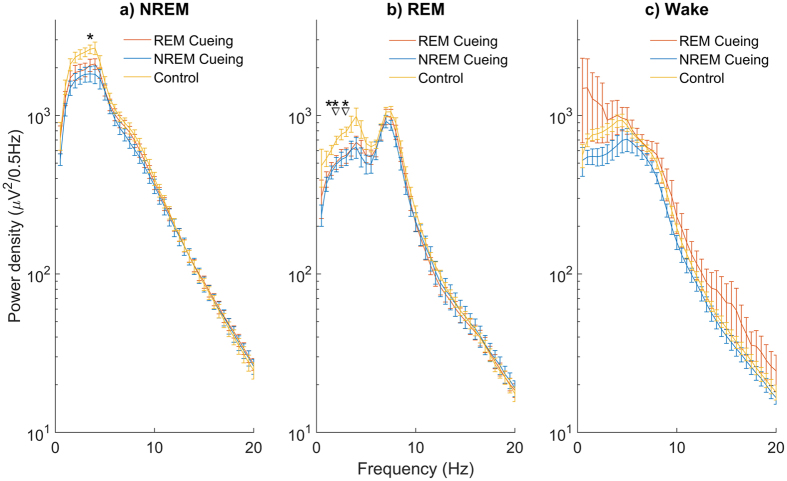
Average spectral power in (**a**) NREM sleep, (**b**) REM sleep, and (**c**) wake states during the 4-hour intervention. Asterisks denote significant differences between control and NREM-cued mice, and triangles denote significant differences between control and REM-cued mice (multivariate general linear models with post-hoc Bonferroni correction). Data are shown as mean ± SEM.

Spectral analysis revealed that NREM- and REM-cued mice showed significantly decreased EEG spectral power within the slow-wave range during NREM and REM sleep (3.5 Hz during NREM sleep and 1.5, 2, and 3 Hz during REM sleep; Fig. 3).

### No group differences in initial learning or context discrimination

**Figure 4:**
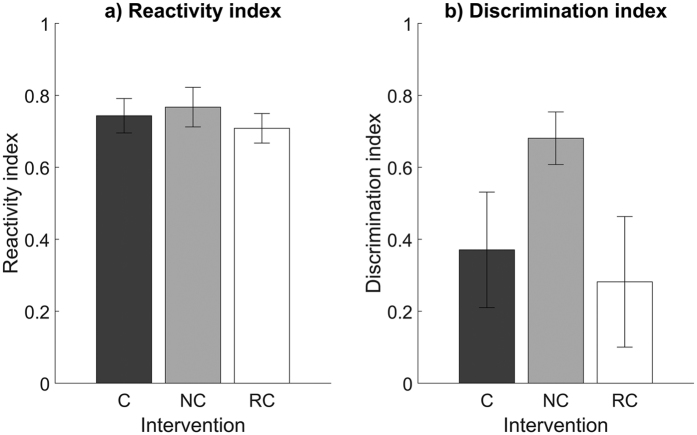
Reactivity and discrimination indices. (**a**) Reactivity to the first foot shock during fear conditioning training (ANOVA, F = 2.14, p = 0.14). (**b**) Discrimination between context B before the auditory CS and context A without the auditory CS (ANOVA, F < 1, p = 0.68). Data are shown as mean ± SEM.

Next, to assess differences in baseline learning during the foot-shock exposure of fear conditioning training, we calculated a reactivity index for each mouse. There were no differences between groups, with all mice showing comparable reactivity to the initial foot-shock (Fig. 4a). We also calculated a discrimination index to measure the similarity in freezing behaviour between exposure to the novel context (context B, 23 hours after training) before the auditory CS and re-exposure to the training context (context A, 28 hours after training) without the auditory CS. NREM-cued mice showed a trend toward greater discrimination between contexts compared with REM-cued and control mice, but these differences did not reach significance (Fig. 4b).

### Auditory cueing during NREM sleep impaired fear memory

**Figure 5:**
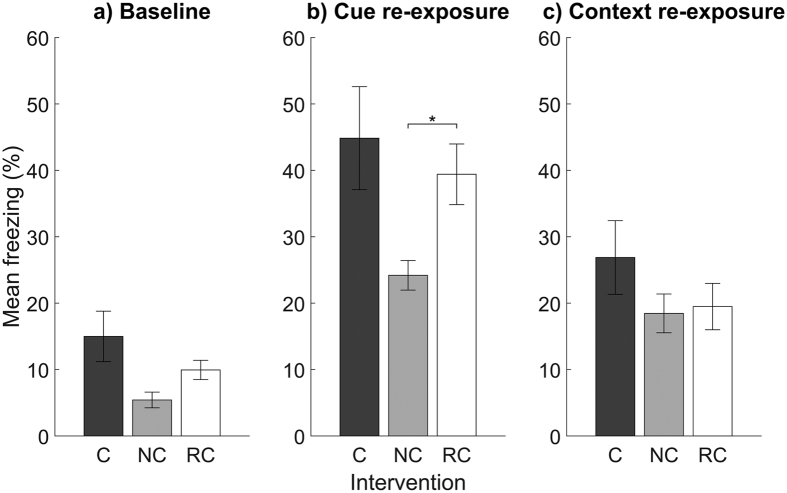
Freezing behaviour for mice in the control (C), NREM-cued (NC), and REM-cued (RC) groups during exposure to (**a**) context B before the auditory CS, (**b**) context B during the auditory CS, and (**c**) context A without the auditory CS. Data are shown as mean ± SEM.

Our primary aim was to identify differences in fear memory after presentation of the CS during NREM or REM sleep. We found that NREM-cued mice showed a trend toward less baseline freezing (i.e., before auditory CS presentation) in context B compared with REM-cued and control mice, but these differences did not reach significance (Table 2, Fig. 5a). However, upon presentation of the auditory CS in context B, NREM-cued mice showed significantly less freezing than REM-cued mice and a trend toward less freezing than control mice. Specifically, NREM-cued mice showed an average of 15.2% less freezing than REM-cued mice and 20.6% less freezing than control mice (Fig. 5b). Finally, there were no group differences in freezing when mice were returned to context A without the auditory CS (Fig. 5c).

## Discussion

Recent studies show that presenting a CS during NREM sleep can enhance, impair, or have no effect on fear memory. In particular, a recent commentary highlights the contradictory results of human and rodent studies^23^. Specifically, two studies with human participants report that re-presenting an olfactory or auditory CS during NREM sleep impairs fear memory consolidation^22^,^23^. By contrast, two studies using rats^13^ and mice^16^ report the opposite effect—that stimulating the olfactory bulb^13^ or re-presenting an olfactory CS^16^ during NREM sleep enhances fear memory consolidation. Therefore, our present finding that presenting an auditory CS during NREM sleep impaired fear memory in mice supports the results of human studies and suggests that fear memories can either be enhanced or impaired depending upon experimental conditions.

What might determine whether fear memories are enhanced or impaired by cueing during sleep? In addition to species differences, other possible explanations include the aversiveness of the shock; the degree of reinforcement contingency (i.e., the proportion of times the CS is paired with the unconditioned stimulus); the timing between training, cueing, and testing; and the frequency of cueing during sleep^23^. However, considering the degree of similarity between the present study and previous rodent studies, these explanations seem implausible. Rather, the discrepancy among studies could be explained by the type of CS. Whereas previous rodent studies^13^,^16^ used olfactory cues, which are processed directly in the piriform cortex^24^, the present study and He *et al*.^21^ used auditory cues, which are processed by multiple nuclei before reaching the primary auditory cortex. This difference in the processing of olfactory versus auditory stimuli could explain the differing effects of cueing during NREM sleep, although the Hauner *et al*.^22^ report of memory impairment after olfactory cueing casts doubt on this explanation.

Another possible explanation for the discrepancy among studies could be related to differences in fear conditioning protocols. Barnes and Wilson^13^ and Rolls *et al*.^16^ employed a delayed conditioning protocol with concurrent presentation of the CS and shock in rodents. He *et al*.^21^ and Hauner *et al*.^22^ also employed a delay conditioning protocol but tested human participants in the same context in which they were trained. In the present study, we used a trace conditioning protocol with a long interval (20 s) between the CS and shock and tested rodents in a novel context. Whereas contextual fear conditioning is classically known as a hippocampal-amygdala-dependent task, cued fear conditioning is primarily amygdala-dependent^25^. However, conditioning with long trace intervals between the CS and shock could potentially place a greater dependence on the hippocampus^26^. Therefore, we speculate that the enhanced fear memory reported by previous rodent studies may be due to greater dependence on the amygdala, whereas the impaired fear memory reported here may be due to greater dependence on the hippocampus due to the use of a long trace interval. This possibility is supported by several lines of evidence. For instance, an early study shows that with a 10-s interval between the CS and unconditioned stimulus, cueing during NREM sleep impairs avoidance fear memory^14^. Also, Wamsley and Antrobus^27^ found that re-presenting a CS during NREM sleep enhances emotional memory among human participants who undergo trace fear conditioning but not those who undergo delay fear conditioning. Furthermore, sleep deprivation consistently impairs contextual learning but only impairs cued learning if the hippocampus is involved, such as in trace conditioning protocols^28^,^29^. Therefore, the conditioning protocol used and its dependency on the hippocampus could critically determine the effect of cueing during sleep on memory consolidation.

A further consideration is how cueing during NREM sleep impairs fear memory, as cueing could interrupt the unfinished consolidation process, destabilize the memory and thereby hinder reconsolidation, or induce new inhibitory learning and memory extinction. In this study, NREM-cued mice showed a trend toward greater discrimination between the conditioned and novel contexts compared with other groups of mice. However, all mice showed similar freezing levels upon re-exposure to the conditioned context. Thus, we speculate that presenting the CS during NREM sleep causes new inhibitory learning and the extinction of conditioned fear memory rather than a weakening of the initial association. As memories of the CS and the context could interfere with each other, extinction of the CS memory could place further emphasis on the conditioned context and hence reduce context generalisation^30^,^31^. Possible future studies that employ cueing outside of the consolidation window, cueing during waking periods, a sub-threshold reminder shock to test for reinstatement, or an examination of spontaneous recovery of fear would be required to fully delineate these processes^32^.

We detected no effect of cueing during REM sleep, which agrees with previous human studies of declarative^17^ or emotional^18^,^19^ memories. This lack of effect during REM sleep could be explained by an absence of slow oscillations and sleep spindles and higher acetylcholine levels during REM sleep as compared with NREM sleep^17^. However, inconsistencies among studies still remain, with some human studies reporting enhanced memory after REM cueing^33^,^34^. To our knowledge, only one other study has assessed REM cueing in rodents, which also reports enhanced avoidance fear memory^15^. The discrepancy between our results and those of the previous rodent study could be due to differences in the type of CS (i.e., auditory CS versus ear shock), which may have differential effects on memory processing during REM sleep. Thus, further investigation is required to fully understand the effect of cueing during REM and NREM sleep in humans and other species. Obvious differences in experimental protocols including the type of memory (e.g., declarative, procedural, or emotional) and CS (e.g., olfactory, auditory, or ear shock) as well as more subtle differences including the timing of stimulus presentation or interval between training and testing likely play a combined and complex role, ultimately affecting regional differences in brain activation during sleep.

Another finding of this study was that NREM/REM cueing had a slight effect on sleep architecture and spectral power. The lack of changes in the total amount of NREM sleep or the number of NREM-to-wake or REM-to-wake transitions indicates that mice were not awoken by auditory cueing. However, the decrease in spectral power in the slow-wave range for both NREM- and REM-cued mice, and the increased number of REM-to-NREM transitions in REM-cued mice suggests that the cueing induced some degree of arousal causing lighter sleep, which could have considerable impact on how memory is consolidated during sleep. However, because auditory arousal thresholds are similar^35^ or even lower in REM than in NREM sleep^36^, but REM-cued mice did not show behavioural changes relative to control mice, it is unlikely that our observed effect of NREM cueing was due to greater arousal. Indeed, a recent study in humans shows that cueing during NREM stage 2 enhances performance in a motor sequence task, suggesting that memories can also be successfully manipulated during lighter sleep^37^.

In summary, this study extends previous literature on the role of sleep in memory processing and shows that cueing during NREM sleep can impair fear memory in rodents. Furthermore, these findings suggest that procedural differences can determine whether cueing during sleep enhances or impairs fear memory, which may be an important consideration when using targeted memory reactivation to treat pathological conditions such as post-traumatic stress disorder.

## Methods

### Animals

All experiments were performed in accordance with the Science Council of Japan’s Guidelines for Proper Conduct of Animal Experiments. Experimental protocols were approved by the Institutional Animal Care and Use Committee at the University of Tsukuba. Thirty male C57BL/6 mice (Jackson Laboratory) were bred in our colony at the University of Tsukuba and maintained on a 12-hour light/dark cycle (lights on 9 am–9 pm) with *ad libitum* access to food and water.

### Surgery

Mice were on average 9 weeks of age on the day of surgery (range: 8–13 weeks). Surgeries were conducted as previously described^38^ using a stereotaxic frame under isoflurane general anaesthesia. Mice were implanted epidurally with four cortical electrodes over the frontal (AP + 1.5 mm, ML ± 1.7 mm) and parietal (AP −3 mm, ML ± 1.7 mm) cortices. EMG signals were recorded from two electrodes placed bilaterally into the trapezius muscles. Electrodes consisted of stainless steel recording screws for EEG and stainless steel Teflon-coated wires for EMG. Electrodes were affixed to the skull using dental cement (Sun Medical; Super-Bond C&B set). After surgery, mice were placed into individual cages and allowed to recover for 6 days, during which they were gently handled for 2 min two to three times daily to become familiarised with experimenter handling. One mouse exhibited a gradual decline after surgery and was therefore removed from further analysis, leaving a total of 29 mice under investigation.

### General fear conditioning procedure and apparatus

Fear conditioning utilises a classical conditioning paradigm that takes advantage of an innate response that can be controlled by learned external stimuli. In rodents, an unconditioned stimulus (e.g., foot shock) produces an unconditioned fear response. If the unconditioned stimulus is repeatedly paired with an emotionally neutral CS (e.g., auditory tone or spatial context), new associative learning occurs, and the presentation of the CS in the absence of the unconditioned stimulus comes to evoke a conditioned fear response^39^.

The auditory CS used in this study was an upward frequency sweep (5–20 kHz, 800 ms, 50 dB^40^), which has successfully been used in previous studies to evoke neuronal responses during sleep^40^,^41^. An additional set of mice, not described in the results of this study, were tested to assess arousal levels during presentation of the auditory CS during REM and NREM sleep after fear conditioning training. These results were used to calibrate the optimum volume of the auditory CS and CS-to-white noise ratio to ensure the CS did not cause arousal during sleep based upon visual inspection of the EEG and EMG signals. White noise (~50 dB) was played throughout the 4-hour sleep intervention to provide background noise^12^. When the auditory CS was presented, the white noise was lowered (CS-to-white noise ratio: 0.3:0.7) so that the overall sound pressure was maintained at ~50 dB. The sound volume was carefully monitored by an audiometer (Koolertron, Shenzhen, China).

The fear conditioning apparatus has previously been described^42^. Briefly, fear conditioning training and contextual fear memory testing (context A) occurred in a stainless-steel chamber (31 × 24 × 21 cm; MED Associates, St. Albans, VT) made of clear acrylic for the front, top, and back sides and aluminium panels for the left and right sides. The floor was a stainless-steel grid with bars (3.2 mm diameter) spaced 7.9 mm apart allowing the delivery of electric shocks. A stainless-steel drop pan under the grid floor was lightly cleaned with 75% ethanol before each experiment, which also provided a background odour. A novel context (context B) was used to test the specificity of freezing behaviour to the auditory CS. For context B, a white plastic floor covered the grid floor, and a grey plastic triangular insert was placed inside the chamber to create artificial left and right sides. The front side consisted of a piece of cardboard with a blue and white rectangular pattern affixed in the centre. Context B was cleaned with water instead of ethanol.

The primary measure of the fear response during fear conditioning testing was freezing, which was defined as behavioural immobility except for movements necessary for respiration^43^. Time spent freezing was calculated offline^1^ using a timer and visual confirmation. The examiner was blind to the group membership of mice.

### Fear conditioning training

Fear conditioning training occurred on day 7 at zeitgeber time (ZT) 2. ZT2 was chosen because it allowed the observation of stable sleep behaviour after switching the light-dark cycle. Trace fear conditioning was performed as previously described^26^. Mice were placed in context A. After 192 s of habituation to the context, an auditory CS (~75 dB) was presented for 20 s, followed by a 20-s trace interval and a 2-s foot shock (0.65 mA). This sequence (234 s) was repeated five times, totalling 19.5 min of training.

### Auditory cueing during sleep

Immediately after fear conditioning training, mice were transferred back to their home cages. Mice were split into three groups that received different types of sleep intervention: NREM cueing, REM cueing, or control (i.e., no auditory CS). In the NREM cueing group the auditory CS was administered when at least 20 seconds of stable NREM was identified. The CS lasted for 10 seconds and a maximum of 3 applications (separated by at least 20 seconds) were allowed for every NREM episode until at total of 5 minutes of the auditory CS was given (as previously reported^16^). For the REM cueing group the auditory CS was administered during every REM episode and was maintained for the duration of the REM episode until a total of 5 minutes of the auditory CS was given. This resulted in at least six periods of NREM or REM sleep accompanied by the auditory CS for all mice (as previously reported^14^,^15^). For control mice, no auditory CS was presented, and white noise was played throughout the intervention. A wake intervention group was not included in the design of this study since CS presentation during wake produces a predictable extinction of fear memory^32^.

### EEG/EMG recording and analysis

Throughout the 4-hour sleep intervention, EEG and EMG recordings were obtained. Signals were digitised at 256 Hz, filtered (band pass 0.5–64 Hz), and visualised in real time using SleepSign Recorder software (Kissei Comtec, Matsumoto, Nagano, Japan). NREM sleep was defined as synchronised high amplitude, low frequency delta waves (0.5–4 Hz) and low EMG activity relative to wakefulness. REM sleep was defined as high frequency theta rhythm (4–9 Hz) and no EMG activity^38^.

EEG/EMG traces were manually scored offline using SleepSign for Animal sleep analysis software (Kissei Comtec, Matsumoto, Nagano, Japan). Signals were further filtered (EEG: band pass 0.5–25 Hz; EMG: band pass 0.5–100 Hz). A 10-s epoch scoring criterion was used. If an epoch contained multiple vigilance states (wake, NREM, or REM), the state with the highest occupancy was assigned. Total amounts of time spent in each vigilance state and the number of transitions between states were compared between groups. EEG signals were also analysed using a Fast Fourier Transform spectral analysis between 0–20 Hz at 0.5-Hz resolution and compared between groups.

### Fear conditioning testing

Twenty-three hours after fear conditioning training, at ZT1, mice were placed in context B. After 3 min of habituation, the auditory CS was continuously played (~75 dB) for 3 min to assess fear responses to the auditory CS. Mice were then returned to their home cages. At ZT6, mice were returned to context A for 5 min to assess fear responses to the training context. Mouse behaviour was video recorded during all tests.

To assess initial learning, a behavioural reactivity index was calculated. For the first shock delivered during fear conditioning training, we measured the velocity of the movement of mice for 2 s before and 2 s during the shock using ImageJ software (National Institutes of Health, Bethesda, Maryland, USA). The reactivity index was calculated as: 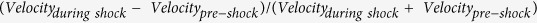
44. To assess the degree of discrimination between context A and context B, a discrimination index was calculated as: 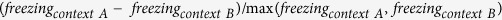
45.

### Statistical analysis

Statistical analysis was performed using R software^46^. Shapiro-Wilk normality tests were used to determine whether data were normally distributed. Differences between groups were tested using analysis of variance (ANOVA) and post-hoc *t*-tests with Bonferroni correction when appropriate. Differences between groups in spectral frequencies during the 4-hour sleep intervention were tested using a multivariate general linear model with post-hoc Bonferroni correction. Bonferroni correction (alpha = 0.017; 0.05/3) was required to avoid inflation of alpha resulting from multiple comparisons between three groups.

## Additional Information

**How to cite this article**: Purple, R. J. *et al*. Auditory conditioned stimulus presentation during NREM sleep impairs fear memory in mice. *Sci. Rep.* **7**, 46247; doi: 10.1038/srep46247 (2017).

**Publisher's note:** Springer Nature remains neutral with regard to jurisdictional claims in published maps and institutional affiliations.

## Figures and Tables

**Table 1:** Group comparisons for measures of sleep architecture during the 4-hour intervention (t-tests with post-hoc Bonferroni correction, α = 0.017).

*Total time in vigilance states*	*ANOVA*	*Group comparison*	*Mean (% time*)	*t*	*df*	*p*
Wake	*F* = 4.22, *p* = 0.026*	C - NC	45.4–38.3	1.43	14.1	0.174
		C - RC	45.4–33.4	2.72	10.5	0.021
		NC - RC	38.3–33.4	1.56	14.7	0.139
NREM	*F* = 3.67, *p* = 0.039*	C - NC	48.4–55.6	−1.65	15.8	0.119
		C - RC	48.4–58.8	−2.63	13.0	0.021
		NC - RC	55.6–58.8	−0.94	16.4	0.363
REM	*F* = 2.14, *p* = 0.138	C - NC	6.2–6.1			
		C - RC	6.2–7.9			
		NC - RC	6.1–7.9			
						
*Vigilance state transitions*	*ANOVA*	*Group comparison*	*Mean (no. of transitions*)	*t*	*df*	*p*
Wake to NREM	*F* = 2.97, *p* = 0.069	C - NC	40.3–33.8			
		C - RC	40.3–29.9			
		NC - RC	33.8–29.9			
Wake to REM	*F* = 0.51, *p* = 0.609	C - NC	0.6–0.3			
		C – RC	0.6–0.3			
		NC - RC	0.3–0.3			
NREM to wake	*F* = 1.95, *p* = 0.163	C - NC	27.0–22.6			
		C - RC	27.0–18.0			
		NC - RC	22.6–18.0			
NREM to REM	*F* = 0.87, *p* = 0.429	C - NC	15.2–14.9			
		C - RC	15.2–17.7			
		NC - RC	14.9–17.7			
REM to wake	*F* = 0.59, *p* = 0.563	C - NC	13.2–10.9			
		C - RC	13.2–11.7			
		NC - RC	10.9–11.7			
REM to NREM	*F* = 3.76, *p* = 0.037*	C - NC	2.3–4.0	−1.30	16.1	0.212
		C - RC	2.3–6.0	−2.98	16.5	0.009*
		NC - RC	4.0–6.0	−1.41	17.9	0.174

Asterisks indicate statistical significance.

**Table 2:** Group comparisons for freezing during the fear memory tests (t-tests with post-hoc Bonferroni correction, α = 0.017).

*Total freezing*	*ANOVA*	*Group comparison*	*Mean (% freezing*)	*t*	*df*	*p*
Context B baseline (3 min before CS)	F = 4.17, p = 0.030*	C - NC	15.0–5.4	2.41	9.5	0.038
		C - RC	15.0–9.9	1.24	10.3	0.241
		NC - RC	5.4–9.9	−2.41	17.3	0.027
Context B CS presentation (3 min during CS)	F = 4.32, p = 0.024*	C - NC	44.8–24.2	2.56	9.3	0.030
		C - RC	44.8–39.4	0.60	13.1	0.556
		NC - RC	24.2–39.4	−3.00	13.1	0.010*
Context A (5 min without CS)	F = 1.25, p = 0.302	C - NC	26.9–18.5			
		C - RC	26.9–19.5			
		NC - RC	18.5–19.5			

Asterisks indicate statistical significance.
